# Efficacy of opioid-free anesthesia in reducing postoperative nausea and vomiting following gynecological laparoscopic surgery: a randomized controlled trial

**DOI:** 10.3389/fmed.2025.1606383

**Published:** 2025-07-17

**Authors:** Yanan Shen, Yuanyuan Wu, Qianqian Tang, Yilong Wang, Wei Ma, Jianwei Wang

**Affiliations:** ^1^Department of Anesthesiology, School of Medicine, International Peace Maternity and Child Health Hospital, Shanghai Jiao Tong University, Shanghai, China; ^2^Shanghai Key Laboratory of Embryo Original Diseases, Shanghai, China

**Keywords:** opioid-free anesthesia, nausea, vomiting, esketamine, dexmedetomidine, transversus abdominis plane block

## Abstract

**Background:**

Postoperative nausea and vomiting (PONV) are a common complication following gynecological laparoscopic surgery, with opioid use being a significant contributing risk factor. Opioid-free anesthesia (OFA) has emerged as an alternative approach to mitigate opioid-related adverse effects. This study aimed to evaluate the efficacy of OFA in reducing PONV and its impact on postoperative recovery.

**Methods:**

This randomized, double-blind, controlled trial enrolled 92 female patients undergoing elective gynecological laparoscopic surgery. The patients were randomized 1:1 into two groups, whereby the OFA group received anesthesia with esketamine (0.3 mg/kg) and dexmedetomidine (0.6 μg/kg), while the control group received conventional opioid-based anesthesia with sufentanil (0.3 μg/kg). Both groups underwent preoperative transversus abdominis plane (TAP) block with ropivacaine (20 mL per side). Standardized perioperative monitoring and analgesic protocols were maintained. The primary outcome was the incidence of PONV within 48 h postoperatively. Secondary outcomes included the Modified Observer’s Assessment of Alertness/Sedation (MOAA/S) score recovery time, postoperative pain scores, antiemetic and analgesic use, and quality of recovery (QoR-15 scores).

**Results:**

On postoperative day 1, PONV incidence was significantly lower in the OFA group (15.2%) compared to the control group (34.8%) (*P* = 0.03), and by postoperative day 2, the difference between the groups was no longer statistically significant (*P* = 0.475). The OFA group exhibited a longer median extubation time (11.0 min vs. 5.5 min, *P* < 0.001) and a prolonged MOAA/S recovery time (45.0 min vs. 40.0 min, *P* = 0.012). Pain scores, postoperative sufentanil consumption, and QoR-15 scores did not differ significantly between groups. No patients in either group required postoperative supplemental analgesics, and three patients in each group received antiemetic treatment solely on the first postoperative day.

**Conclusion:**

Opioid-free anesthesia incorporating TAP block may reduce early PONV following gynecological laparoscopic surgery while maintaining adequate pain control and overall recovery quality.

## Introduction

Postoperative nausea and vomiting (PONV) are among the most common complications following surgery, with an incidence ranging from 20% to 37% in recent studies and increasing to as high as 80% in patients with multiple high-risk factors, such as female sex, a history of motion sickness or previous PONV, non-smoking status, and postoperative opioid use, particularly in those undergoing general anesthesia (1, 2). The simplified Apfel risk score is widely used to assess PONV risk in hospitalized patients, with individuals presenting three or four risk factors exhibiting a significantly higher incidence than those at lower risk (3).

Opioids are potent analgesics that enhance surgical and anesthesia safety by suppressing sympathetic and motor nerve responses, thereby reducing intraoperative sedative requirements. However, their use is associated with adverse effects such as PONV, respiratory depression, pruritus, and constipation (4). Advances in clinical anesthesia have led to the development of opioid-free anesthesia (OFA), an alternative anesthetic approach that avoids opioid-related complications. OFA primarily incorporates non-opioid analgesics, including α2-adrenergic receptor agonists, lidocaine, *N*-methyl-D-aspartate (NMDA) receptor antagonists, and non-steroidal anti-inflammatory drugs, in combination with regional blockade, to achieve effective analgesia (4, 5).

In patients undergoing gynecological laparoscopic surgery, perioperative opioid use has been shown to significantly increase the incidence of PONV, negatively impact postoperative recovery, and prolong hospital stay (6). Previous studies have demonstrated the feasibility of OFA in various surgical procedures, including gynecological laparoscopy (1, 7, 8). However, its effectiveness in reducing PONV in this patient population remains inconclusive (7).

Given the high risk of PONV in this setting, we further optimized the OFA protocol based on prior research. This study aimed to determine whether OFA reduced the incidence of PONV in patients undergoing gynecological laparoscopic surgery and to evaluate its impact on postoperative recovery. The findings are expected to provide clinical evidence supporting the broader application of OFA in high-risk surgical populations.

## Materials and methods

### Ethics approval and consent

This randomized controlled trial was approved by the Ethics Committee of the International Peace Maternity and Child Health Hospital, affiliated with Shanghai Jiao Tong University (Approval Number: GKLW-A-2024-034-01), and is registered in the Chinese Clinical Trial Registry (Registration Number: ChiCTR2400088413)^1^. The study was conducted in accordance with the Declaration of Helsinki and the Consolidated Standards of Reporting Trials (CONSORT). Written informed consent was obtained from all patients prior to participation.

### Inclusion and exclusion criteria

The study inclusion criteria were female patients aged 18–65 years with an American Society of Anesthesiologists (ASA) status of class I or II who were scheduled to undergo elective gynecological laparoscopic surgery for benign tumors. Patients were excluded if they were pregnant, had an ASA classification of III or higher, required emergency surgery, had a psychiatric illness, or had a body mass index (BMI) greater than 30 kg/m^2^. Additional exclusion criteria were a known allergy to any of the anesthetic agents used in this study or the need for intraoperative conversion to laparotomy.

### Randomization and blinding procedures

**FIGURE 1:**
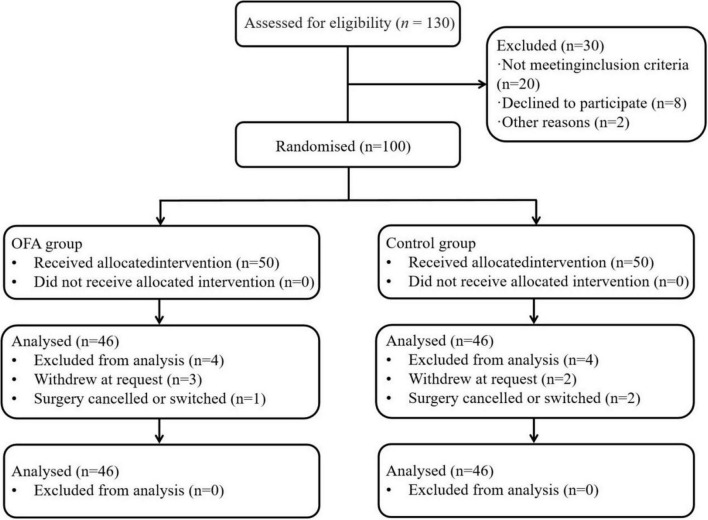
Flow diagram of the trial design.

Participants were randomly assigned in a 1:1 ratio to either the control group (Group A) or the OFA group (Group B) using an online randomization tool (Sealed Envelope)^2^ (Figure 1). Block randomization with block sizes of 2 and 4 was performed following the methodology described by Feng et al. (9).

On the day before surgery, a clinical coordinator assessed each participant and obtained written informed consent. The randomization result was concealed in an opaque envelope, which was opened by the coordinator only after the patient entered the operating room. The coordinator, who was not involved in data collection or postoperative follow-up, prepared the trial medications according to the assigned group, which were then given to the anesthesiologists for anesthesia induction.

Postoperative follow-up was conducted by two independent data collectors who were blinded to group allocation, did not participate in anesthetic management or patient care, and had no access to anesthesia records. These data collectors recorded and summarized the study data. Apart from the clinical coordinator, all patients, surgeons, anesthesiologists, and researchers responsible for outcome evaluation remained blinded to group allocation. All participants underwent standardized anesthetic management and continuous intraoperative monitoring to ensure consistency in perioperative care.

### Anesthesia management and intervention protocols

Upon entering the operating room, all patients underwent routine monitoring, including electrocardiography, non-invasive blood pressure measurement, pulse oximetry, and bispectral index (BIS) monitoring. Intravenous sedation was initiated with midazolam 2 mg, followed by skin disinfection. The patients then underwent bilateral ultrasound-guided transversus abdominis plane (TAP) blocks via the lateral approach, as previously described (10), with an injection of 20 ml of 0.25% ropivacaine per side on both sides.

Anesthesia induction varied between groups. In the control group, induction was performed with intravenous propofol (1.5–2 mg/kg) and sufentanil (0.3 μg/kg); meanwhile, in the OFA group, induction consisted of intravenous propofol (1.5–2 mg/kg) plus esketamine (0.3 mg/kg) with or without dexmedetomidine (0.6 μg/kg) (9) infused over 10 min. Both groups received rocuronium (0.6 mg/kg) for muscle relaxation. Following intubation, dexamethasone (5 mg) and ondansetron (4 mg) were administered prophylactically to reduce the risk of PONV, and propacetamol (1 g) was administered.

Anesthesia was maintained using a target-controlled infusion of propofol at 1 μg/ml, supplemented with inhaled sevoflurane to maintain BIS values between 40 and 60. Hemodynamic stability was ensured by keeping blood pressure and heart rate fluctuations within ± 20% of baseline values. Intraoperative analgesia was adjusted according to surgical demands. In the control group, sufentanil (0.1 μg/kg) was administered intermittently, while the OFA group received esketamine (0.1 mg/kg) to maintain an intraoperative surgical pleth index (SPI) between 20 and 50. Muscle relaxation was maintained with intermittent administration of rocuronium (0.15–0.2 mg/kg). In cases of bradycardia (heart rate < 45 beats/min), atropine (0.5 mg) was administered intravenously. Hypotension, defined as a mean arterial pressure (MAP) < 60 mmHg or a > 20% decrease from baseline, was managed with intravenous ephedrine (10 mg), while hypertension, defined as MAP > 120 mmHg or a > 20% increase above baseline, was treated with intravenous nicardipine (0.1 mg). After surgery, once the patients became awake and their BIS exceeded 80, sugammadex (2 mg/kg) was administered intravenously to reverse neuromuscular blockade, and the endotracheal tube was removed.

Postoperative analgesia comprised several methods with intravenous administration of 1 g propacetamol on postoperative days one and two. Patient-controlled intravenous analgesia was provided with sufentanil (1 μg/ml), delivered at a continuous infusion rate of 1 ml/h, with a bolus dose of 2 ml and a lockout interval of 10 min. Patients were instructed to use patient-controlled intravenous analgesia as needed to manage postoperative pain. Postoperative pain was assessed using the Numerical Rating Scale (NRS), and nausea and vomiting were assessed using the Visual Analogue Scale (VAS). Additional analgesia (NRS ≥ 4) with intravenous sufentanil (5 μg) and antiemetic therapy (VAS ≥ 4) with intravenous ondansetron (4 mg) was administered.

### Trial outcomes

The primary outcome was the incidence of PONV within 48 h after surgery, defined as any occurrence of nausea, retching, or vomiting. PONV incidence was assessed at 24 and 48 h postoperatively by an independent assessor in the ward (9). Subjects were asked to rate their PONV episodes in the preceding 24 h on the simplified PONV impact scale (consisting of two questions: Q1. Have you had vomiting or retching? Q2. Have you experienced nausea? If yes, has the feeling of nausea interfered with activities of daily living?) (11).

Secondary outcomes included the time required to achieve a Modified Observer’s Assessment of Alertness/Sedation (MOAA/S) score greater than 4 in the post-anesthesia care unit (PACU), the incidence of prolonged PACU recovery exceeding 2 h, nausea and vomiting scores, pain scores, and the frequency of additional analgesic and antiemetic administration. Additional secondary endpoints included Quality of Recovery-15 (QoR-15) scores and the total postoperative dose of sufentanil administered.

### Statistical analysis

The incidence of PONV after conventional opioid-based anesthesia is reported to be 41.7% (12), with opioid reduction potentially decreasing this incidence to 11.4%. Using an α level of 0.05, a power (1-β) of 0.8, and an allocation ratio of 1:1, the required sample size was calculated using R software based on the methodology described by Chow et al. (13), which yielded a sample size of 40 patients per group. To account for an anticipated 20% dropout rate, the total sample size was set at 100 patients.

The data are expressed as mean ± standard deviation (SD), median with interquartile range, or number with percentage, as appropriate. The normality of continuous variables was assessed using the Shapiro-Wilk test. Normally distributed data were analyzed using independent sample *t*-tests, while non-normally distributed data were compared using the Mann-Whitney U test. Categorical variables were analyzed using the chi-square test, chi-square test with Yates’ continuity correction, or Fisher’s exact test, as appropriate. These statistical analyses were performed using SPSS software (version 19.0; IBM SPSS, Chicago, IL, United States), with two-sided *p*-values < 0.05 considered statistically significant.

## Results

### Patient enrollment and baseline characteristics

A total of 100 patients were initially enrolled in the study; however, three patients did not undergo their planned surgery, and five withdrew their informed consent. As a result, 92 patients completed the study and postoperative follow-up. The baseline characteristics of the participants are presented in Table 1, and data analysis indicated that the two groups were comparable.

**TABLE 1:** Baseline characteristics of the included patients.

Variables	Overall (*n* = 92)	Control group (*n* = 46)	OFA group (*n* = 46)	*P*-value
Age (years), mean (SD)	44.75 (8.69)	44.57 (6.94)	44.93 (10.22)	0.840
Height (cm), mean (SD)	159.96 (5.10)	160.85 (5.22)	159.07 (4.86)	0.094
Weight (kg), mean (SD)	60.31 (8.53)	60.50 (8.37)	60.12 (8.78)	0.832
BMI (kg/m), mean (SD)	23.57 (3.10)	23.37 (2.90)	23.76 (3.30)	0.539
History of smoking, *n* (%)	3 (3.3)	1 (2.2)	2 (4.3)	1.000
History of motion sickness, *n* (%)	26 (28.3)	10 (21.7)	16 (34.8)	0.165

### Primary outcome

On postoperative day 1, 16 patients in the control group experienced PONV, whereas only seven patients in the OFA group were affected, representing a 17.6% reduction in PONV incidence in the OFA group compared with the control group (*P* = 0.03) (Table 2). By postoperative day 2, the number of patients experiencing PONV had decreased in both groups, and the difference between groups was no longer statistically significant (*P* = 0.475) (Table 3).

**TABLE 2:** Patients’ outcomes on postoperative day 1.

Variables	Overall (*n* = 92)	Control group (*n* = 46)	OFA group (*n* = 46)	*P*-value
PONV incidence, *n* (%)	23(25.0)	16 (34.8)	7 (15.2)	0.030
Nausea and vomiting score [mean (SD)]	0.73 (1.48)	0.93 (1.50)	0.52 (1.46)	0.183
Nausea, *n* (%)	23(25.0)	16 (34.8)	7 (15.2)	0.030
Retching, *n* (%)	16 (17.4)	13 (28.3)	3 (6.5)	0.006
Vomiting, *n* (%)	15 (16.3)	12 (26.1)	3 (6.5)	0.011
Nausea affecting daily life, *n* (%)	0 (0.0)	0 (0.0)	0 (0.0)	NA
PCIA consumption (ml) [median (IQR)]	27.05 (23.00, 29.83)	27.25 (22.92, 30.20)	26.95 (23.42, 29.73)	0.623
QoR-15 score [median (IQR)]	133.00 (127.75, 140.00)	131.50 (125.00, 140.75)	133.50 (129.25, 139.00)	0.360
VAS (at rest) [median (IQR)]	2.00 (0.00, 3.25)	2.00 (1.00, 4.00)	2.00 (0.00, 3.00)	0.445
VAS (during movement) [median (IQR)]	3.00 (2.00, 5.00)	4.00 (2.00, 5.00)	3.00 (2.00, 4.00)	0.395
Nightmares, *n* (%)	0 (0.0)	0 (0.0)	0 (0.0)	NA
Dizziness, *n* (%)	4 (4.3)	3 (6.5)	1 (2.2)	0.609
Hallucinations, *n* (%)	2 (2.2)	2 (4.3)	0 (0.0)	0.475
Required additional analgesia, *n* (%)	0 (0.0)	0 (0.0)	0 (0.0)	NA
Required additional antiemetics, *n* (%)	3 (6.5)	3 (6.5)	1 (2.2)	0.617

**TABLE 3:** Patients’ outcomes on postoperative day 2.

Variables	Overall (*n* = 92)	Control group (*n* = 46)	OFA group (*n* = 46)	*P*-value
PONV incidence, *n* (%)	2 (2.2)	2 (4.3)	0 (0.0)	0.475
Nausea and vomiting score [mean (SD)]	0.17 (0.82)	0.35 (1.14)	0.00 (0.00)	0.041
Nausea, *n* (%)	2 (2.2)	2 (4.3)	0 (0.0)	0.475
Retching, *n* (%)	1 (1.1)	1 (2.2)	0 (0.0)	1.000
Vomiting, *n* (%)	0 (0.0)	0 (0.0)	0 (0.0)	NA
Nausea affecting daily life, *n* (%)	1 (1.1)	1 (2.2)	0 (0.0)	1.000
PCIA consumption (ml) [median (IQR)]	55.10 (50.75, 59.62)	56.15 (51.75, 59.62)	53.10 (49.75, 59.38)	0.563
QoR-15 score [median (IQR)]	144.00 (142.00, 148.00)	144.00 (142.00, 148.00)	144.00 (143.00, 147.75)	0.897
VAS (at rest) [median (IQR)]	0.00 (0.00, 2.00)	0.50 (0.00, 2.00)	0.00 (0.00, 2.00)	0.253
VAS (during movement) [median (IQR)]	2.00 (1.00, 3.00)	2.00 (1.00, 2.75)	2.00 (1.00, 3.00)	0.936
Nightmares, *n* (%)	0 (0.0)	0 (0.0)	0 (0.0)	NA
Dizziness, *n* (%)	1 (1.1)	1 (2.2)	0 (0.0)	1.000
Hallucinations, *n* (%)	0 (0.0)	0 (0.0)	0 (0.0)	NA
Additional analgesia required, *n* (%)	0 (0.0)	0 (0.0)	0 (0.0)	NA
Additional antiemetics required, *n* (%)	0 (0.0)	0 (0.0)	0 (0.0)	NA

### Secondary outcomes

Patients in the OFA group exhibited significantly prolonged sedation recovery times in the PACU compared with those in the control group, as indicated by a longer time to achieve an MOAA/S score greater than 4 (*P* = 0.012). However, no patient in either group experienced prolonged PACU recovery exceeding 2 h (Table 4).

**TABLE 4:** Patient performance in the post-anesthesia care unit (PACU).

Variables	Overall (*n* = 92)	Control group (*n* = 46)	OFA group (*n* = 46)	*P*-value
Nausea, *n* (%)	3 (3.3)	0 (0.0)	3 (6.5)	0.240
Retching, *n* (%)	3(3.3)	0 (0.0)	3 (6.5)	0.240
Vomiting, *n* (%)	2 (2.2)	0 (0.0)	2 (4.3)	0.475
Time to MOAA/S > 4 (min) [median (IQR)]	40.00 (30.00, 50.00)	40.00 (25.00, 45.00)	45.00 (35.00, 55.00)	0.012
VAS score [median (IQR)]	3.00 (2.00, 4.00)	3.00 (2.00, 4.00)	3.00 (2.00, 4.00)	0.491
Prolonged recovery, *n* (%)	0 (0.0)	0 (0.0)	0 (0.0)	NA

Data are presented as median (interquartile range) or number (%). NA, not applicable.

Nausea and vomiting scores were significantly lower in the OFA group on postoperative day 2 (*P* = 0.041), and the symptoms remained mild in both groups at all postoperative time points (Tables 2, 3). Only three patients in the OFA group required additional antiemetic medication on postoperative day 1, and symptom relief was achieved (Table 2).

There were no significant differences between the two groups regarding pain scores or postoperative sufentanil consumption, and no patients in either group required additional analgesic medications (Tables 2, 3). In addition, postoperative QoR-15 scores also did not differ significantly between groups, with both groups achieving excellent QoR-15 scores (≥ 136) by postoperative day 2 (Tables 2, 3).

### Intraoperative anesthetic management and adverse events

Apart from differences in the use of dexmedetomidine, sufentanil, and esketamine, no significant differences were observed between groups regarding intraoperative anesthetic doses, duration of surgery, or the incidence of adverse events (Table 5). However, extubation time was significantly prolonged in the OFA group compared with the control group (*P* < 0.001) (Table 5).

**TABLE 5:** Intraoperative medication use, surgical duration, extubation time, and adverse events.

Variables	Overall (*n* = 92)	Control group (*n* = 46)	OFA group (*n* = 46)	*P*-value
Rocuronium (mg) [median (IQR)]	80.00 (70.00, 100.00)	75.00 (66.25, 100.00)	100.00 (70.00, 107.50)	0.143
Propofol (mg) [median (IQR)]	337.50 (240.00, 480.00)	347.50 (300.00, 477.50)	325.00 (202.50, 467.50)	0.342
Sevoflurane (ml) [median (IQR)]	25.00 (19.25, 28.75)	25.00 (19.00, 30.00)	22.00 (20.00, 27.00)	0.158
Dexmedetomidine (go) [median (IQR)]	NA	NA	26.50 (25.00, 30.00)	NA
Sufentanil (μg) [median (IQR)]	NA	20.00 (20.00, 25.00)	NA	NA
Esketamine (mg) [median (IQR)]	NA	NA	20.00 (18.30, 25.00)	NA
Surgical duration (min) [median (IQR)]	115.00 (82.50, 142.50)	110.00 (80.00, 145.00)	120.00 (85.00, 145.00)	0.412
Extubation time (min) [median (IQR)]	8.00 (5.00, 11.00)	5.50 (4.00, 7.00)	11.00 (8.00, 13.50)	< 0.001
Hypotension, *n* (%)	1 (1.1)	1 (2.2)	0 (0.0)	1.000
Bradycardia, *n* (%)	5 (5.4)	2 (4.3)	3 (6.5)	1.000
Hypertension, *n* (%)	6 (6.5)	2 (4.3)	4 (8.7)	0.673
Tachycardia, *n* (%)	0 (0.0)	0 (0.0)	0 (0.0)	NA

## Discussion

In this study of patients undergoing gynecological laparoscopic surgery, all patients in the OFA group completed the procedure safely and successfully. The results indicated that compared with conventional opioid-based anesthesia, OFA did not increase postoperative pain intensity or analgesic consumption, nor negatively impact the quality of patient recovery. Moreover, patients in the OFA group experienced a statistically significant reduction in PONV.

Previous studies have demonstrated that OFA is a safe and reliable anesthetic approach for gynecological laparoscopic surgery and may contribute to improved postoperative sleep quality (6, 7). A randomized controlled trial conducted using Enhanced Recovery After Surgery (ERAS) protocol for gynecological laparoscopic surgery found that the analgesic efficacy of esketamine combined with dexmedetomidine was comparable to that of remifentanil, while OFA significantly reduced the incidence of PONV on the first postoperative day (6). In contrast, Massoth et al. (6) reported that although OFA provided effective intraoperative analgesia and anesthetic stability, it did not significantly reduce the incidence of PONV in patients undergoing gynecological laparoscopic surgery. In this present study, the incidence of PONV on the first postoperative day was 15.2% in the OFA group, significantly lower than the 34.8% observed in the control group, and several factors might have accounted for this discrepancy. First, regional blockade, a key component of OFA, was not utilized in the study by Massoth et al. (6). Since regional blockade reduces intraoperative opioid requirements and postoperative pain intensity, its absence may have contributed to the higher incidence of PONV in their study (6, 9). In the present study, both groups received preoperative TAP blockade; however, patients in the OFA group may have derived greater benefit from this intervention, possibly leading to a more pronounced reduction in PONV. Second, patients in the study by Massoth et al. (6) received higher intraoperative doses of sufentanil, dexmedetomidine, and esketamine, along with a longer duration of anesthesia, all of which could influence PONV incidence. The observed reduction in PONV with OFA was primarily evident on the first postoperative day, while no significant difference between groups was noted on postoperative day two. This finding aligns with the results reported by Feng et al. (9), further supporting the potential of OFA to mitigate early PONV in this patient population. Third, the observed convergence in PONV incidence by postoperative day 2 reflects contemporary recovery trajectories. Modern anesthetic agents and minimally invasive techniques have reduced surgical stress duration. As most inflammatory markers normalize within 48 h after laparoscopy (14–16), differences in anesthesia-induced PONV risk naturally become attenuated during this recovery phase.

Previous studies on OFA have reported conflicting results regarding its impact on reducing postoperative opioid consumption and improving postoperative recovery. In the present study, OFA did not demonstrate a significant advantage in either of these aspects, and this lack of difference may be attributed to the use of TAP block in both groups, which effectively alleviated postoperative pain. Although regional anesthesia is a key component of OFA, it has rarely been incorporated into previous OFA studies. In this study, the combination of TAP block, esketamine, and a single dose of dexmedetomidine simplified the implementation of OFA. As a result, in female patients undergoing laparoscopic surgery, a procedure associated with a high incidence of PONV, we observed that the incidence of PONV was effectively reduced.

Dexmedetomidine is an α2-adrenergic receptor agonist that provides sedative, anxiolytic, sympatholytic, and analgesic effects. A meta-analysis of 21 randomized controlled trials conducted by Grape et al. (17) demonstrated that dexmedetomidine could effectively replace remifentanil for intraoperative analgesia during general anesthesia, reducing postoperative analgesic requirements. Consequently, many OFA-related studies have utilized continuous intraoperative infusion of dexmedetomidine (5, 18). However, a recent clinical study by Beloeil et al. (19) reported that while dexmedetomidine possesses analgesic properties, continuous infusion was associated with severe adverse events in some patients, including intraoperative bradycardia and postoperative hypoxemia, leading to the early termination of their trial. In our present study, the OFA regimen was simplified by administering a preoperative TAP block combined with a single dose of dexmedetomidine during anesthetic induction. This approach effectively met intraoperative analgesic requirements while reducing the risk of adverse events. However, extubation time and the time to sedation recovery (MOAA/S > 4) were significantly prolonged in the OFA group, which may be related to the sedative effect of dexmedetomidine. Previous studies have suggested that remifentanil can induce postoperative hyperalgesia, resulting in increased pain intensity and analgesic consumption (20, 21). To mitigate this effect, we followed the protocols described by Massoth et al. (6), Zhang et al. (22) utilizing intraoperative sufentanil instead of remifentanil for analgesic maintenance.

Quality of recovery-15 is a validated assessment tool for postoperative recovery quality, developed as a simplified version of the QoR-40 scale (23). A meta-analysis of 26 clinical trials conducted by Myles et al. (24) confirmed that QoR-15 is a valid, reliable, and responsive measure for evaluating postoperative recovery in surgical patients. In a previous OFA study involving breast cancer surgery, patients in the OFA group had significantly higher QoR-15 scores at 24 h postoperatively compared with those in the control group, suggesting that OFA may enhance early postoperative recovery quality. However, in the present study, no significant difference in QoR-15 scores was observed between the two groups on postoperative day one. This discrepancy may be attributed to variations in surgical procedures and differences in OFA protocols. Additionally, QoR-15 scores in both groups reached an excellent level (≥ 136) by postoperative day 2 (25), indicating that mild PONV did not adversely affect overall patient recovery.

This study had several limitations that should be acknowledged. First, regional blockade, an important component of OFA, was performed preoperatively in both groups, and this might have contributed to reduced intraoperative sufentanil consumption in the control group and influenced PONV incidence. Second, there was no standardized OFA protocol, and the regimen used in this study was adapted from previous literature, highlighting the need for further protocol optimization. Lastly, this study was conducted at a single center with a relatively small sample size, indicating the need for further validation through larger multicenter studies.

In conclusion, this study explored an OFA protocol incorporating a TAP block, and this approach was found to be simple to implement and effectively reduced PONV in patients undergoing gynecological laparoscopic surgery while maintaining intraoperative safety and anesthetic stability. Additionally, our approach did not increase postoperative pain intensity or negatively impact patient recovery. Therefore, OFA could be a feasible anesthetic strategy for gynecological laparoscopic procedures and potentially beneficial for patients at high risk of PONV.

## Data Availability

The original contributions presented in this study are included in this article/supplementary material, further inquiries can be directed to the corresponding authors.

## Footnotes

1
https://www.chictr.org.cn/showproj.html?proj=239994

2
https://www.sealedenvelope.com
